# The value of cytoplasmic Y-box-binding protein 1 as a prognostic marker for breast cancer in Korean

**DOI:** 10.1007/s12282-015-0625-8

**Published:** 2015-07-21

**Authors:** Anbok Lee, Juhyun Woo, Heejung Park, Sun Hee Sung, Ju-Young Seoh, Woosung Lim, Byung-In Moon

**Affiliations:** 1 Department of Surgery, Busan Paik Hospital, Inje University, College of Medicine, Busan, Korea; 2 Department of Surgery, School of Medicine, Mokdong Hospital, Ewha Womans University, Seoul, Korea; 3 Department of Pathology, School of Medicine, Mokdong Hospital, Ewha Womans University, Seoul, Korea; 4 Department of Microbiology, School of Medicine, Ewha Womans University, Seoul, Korea

**Keywords:** YBX1 protein, Human, Cytoplasm, Prognosis, Breast neoplasm

## Abstract

**Background:**

The human Y-box-binding protein 1 (YB-1) is a member of the DNA/RNA-binding family of proteins that regulates transcription and translation of genes. Previous studies suggest that YB-1 may have an oncogenic role in various cancers. In this study, we evaluate the prognostic value of cytoplasmic YB-1 with respect to breast cancer.

**Methods:**

Immunohistochemical staining study was performed with YB-1 using tissue block from 233 patients with invasive ductal carcinoma. Patients were divided into two groups according to expression of cytoplasmic YB-1 in tumor cell (high versus low). The relationship between the expression of YB-1, clinicopathological characteristics and breast cancer prognosis was analyzed.

**Results:**

Hormone receptor negativity, worse histologic and nuclear grade, high tumor stage, lymphovascular invasion and high Ki67 (≥14 %) were related with the increased expression of cytoplasmic YB-1 in tumor cell (*p* < 0.05). Although there was no significant difference in relapse-free survival (RFS) between the two groups (*p* = 0.412), difference in overall survival (OS) was statistically significant (*p* = 0.035). In multivariate analysis for OS, YB-1 was an independent prognostic factor (*p* = 0.043).

**Conclusion:**

This suggests that the increased expression of cytoplasmic YB-1 in tumor cells can be regarded as an independent prognostic factor for breast cancer, related to poor prognostics. Expression of cytoplasmic YB-1 in cancer cell could be used as an independent prognostic marker for predicting OS in breast cancer.

## Introduction

Breast cancer is the most common cancer for women and its incidence is gradually increasing [1]. It is a kind of heterogeneous disease due to its broad spectrum of morphological features, clinical manifestations, and treatment responses [2]. Therefore, it needs various makers for predicting prognosis and many previous studies have searched for new markers for prognosis and treatment in breast cancer.

Recently, the human Y-box-binding protein 1 (YB-1) has been suggested as a new prognostic marker in many studies. YB-1 is a nucleic acid binding protein, which is located in chromosome 1p34. The process that cold shock domain of YB-1 binds with the Y-box in the gene promoter regulates gene transcription and translation. YB-1 is also known to be involved in DNA repair, splicing, stabilization, cell proliferation and regeneration [3–5].

Several studies have reported on the oncogenic role of YB-1 and mainly focused on the nuclear expression of YB-1 in tumor cell. Nuclear expression of YB-1 in tumor cells is known to be associated with poor prognosis in various types of cancer [6–9]. It has been also reported to be involved in multidrug resistance. Translocation of YB-1 into nucleus activates the transcription of multidrug resistance (MDR) 1 gene and the expression of P-glycoprotein, subsequently producing drug resistance [10]. In matter of breast cancer, YB-1 has been reported to induce human epidermal growth factor receptor (HER)-2. The nuclear expression of YB-1 has been introduced to be related with the negative expression of estrogen receptor (ER) and progesterone receptor (PR) [11]. Furthermore, according to previous report, it has been also known that cytoplasmic expression of YB-1 is related with cancer prognosis [12].

Based on these considerations, this study aims to evaluate the significance of cytoplasmic YB-1 expression for prognosis prediction in breast cancer.

## Methods

### Patient selection

Between January 2003 and December 2008, a total of 538 patients underwent breast cancer surgery at the Department of Surgery, Ewha Womans University Mokdong Hospital. Among these patients, 233 patients were finally enrolled in this study. Patient exclusion criteria were as follows: (1) male, (2) patient who received neoadjuvant chemotherapy, (3) ductal carcinoma in situ, (4) distant metastasis at the time of diagnosis, (5) 2+ for HER2 by immunohistochemistry (IHC), (6) death unrelated with breast cancer, (7) patient with other concomitant cancer (except papillary thyroid cancer, cervical intraepithelial neoplasia:CIN, non-melanoma skin cancer) and (8) patient with no paraffin block or inappropriate paraffin block for study. Molecular subtype was defined as follows: luminal A (ER+ and/or PR+, Ki67 < 20 and HER2−), luminal B (ER+ and/or PR+, Ki67 ≥ 20 and/or HER2+), HER2+ enrich (ER−, PR− and HER2+) and triple negative breast cancer (TNBC) (ER−, PR− and HER2−). Cases with 2+ score were excluded from this study because fluorescent in situ hybridization (FISH) was not performed at our institute prior to 2006.

This study was approved by the Institutional Review Board of Ewha Womans University Mokdong Hospital (IRB approval number, 12-16A-18).

### Immunohistochemical staining

Formalin-fixed paraffin sections (2.5 µm) of tumor tissues were deparaffinized with xylene and rehydrated in graded alcohols. Immunohistochemical staining was done with the Dako Envision system, which uses dextran polymers conjugated with horseradish peroxidase (Dako, Carpinteria, CA, USA). For antigen retrieval, the slides in a target retrieval solution (pH 6.0; DAKO) were boiled for 10 min in a microwave oven. Tissue sections were incubated in a humidified box at 4 °C overnight with a primary antibody (Ab) against YB-1 (1:100 dilutions, Novus USA). After incubation, the sections were treated with an Envision+System-HRP secondary Ab (DaKo). Color development was conducted using 3-amino-9-ethylcarbazole (AEC) (DAKO) as a chromogen. Finally, all sections were counterstained with hematoxylin (DaKo). Negative control sections were treated in the same manner except if they were incubated with Tris-buffered saline without primary Ab.

### Interpretation

**Fig. 1:**
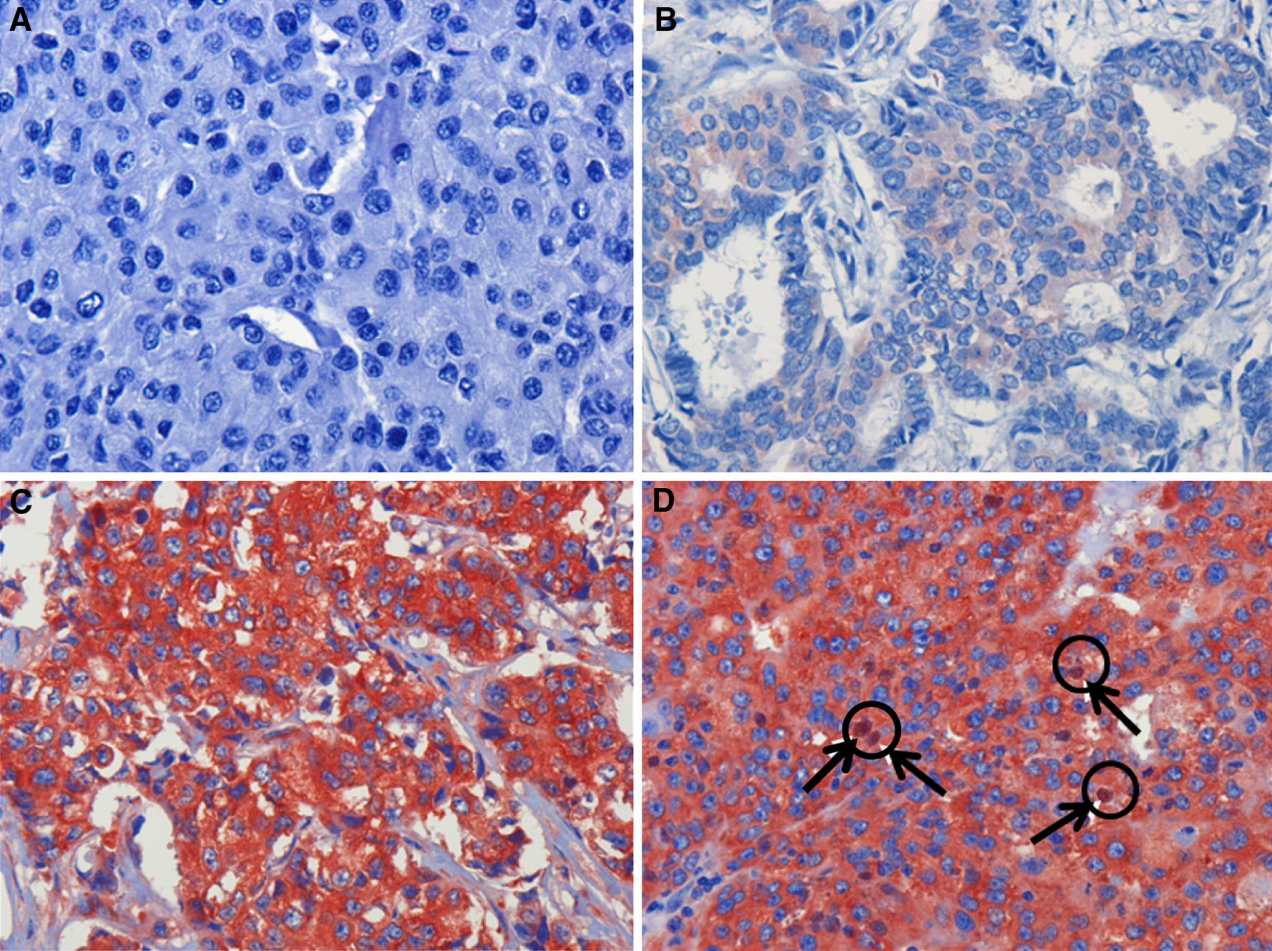
Four different patterns of microscopic findings of immunohistochemical staining for YB-1 expression in showing this study. **a** YB-1 was not expressed in cytoplasm and nucleus. **b** YB-1 was expressed weakly in cytoplasm. **c** YB-1 was expressed strongly in cytoplasm but not in the nucleus. **d** YB-1 was expressed strongly in both cytoplasm and nucleus (*black arrows* and *circles*)

To evaluate the expression for YB-1, two pathologists independently reviewed the slides using an immunoreactivity score (IRS) without any patient information. IRS was evaluated by multiplying the intensity score by percentage of positive tumor cells. The staining results were scored by (1) intensity scores: 0 (no staining), 1 (weak), 2 (moderate), 3(strong); and (2) percentage of positive tumor cells: 0 (0–10 %), 1 (11–25 %), 2 (26–50 %), 3 (51–75 %), 4 (76–100 %). Patients were divided into two groups according to IRS. Scores 0–4 were considered as the low expression group (low group) and 5–12 were considered as the high expression group (high group) (Fig. 1). We determined score which showed best statistical results for prognostic factors and prognosis as cutoff value. Expressions of Hormone receptor and HER2+ were determined based on IHC profiles. ER/PR positivity was defined when more than 10 % of cancer cells were stained with more than 1+ of intensity. In respect to c-erbB2 expression, score of 3+ was regarded as positive and scores of 0, 1+ were regarded as negative.

### Statistical analysis

Pearson’s Chi-square test and Fisher’s exact test were used to compare patient’s clinicopathologic characteristics. Relapse-free survival (RFS) was defined as a length of time from surgery to relapse of cancer and overall survival (OS) was defined as from surgery to breast cancer related death and analyzed by the Kaplan–Meier method. Univariate analysis was performed using Log-rank test and multivariate analysis was performed using Cox’s proportional hazard model with 95 % confidence interval (CI). The mean value comparison of chemotherapeutic agents was conducted by the Mann–Whitney test. All statistical tests were performed by SPSS (version 18, SPSS Inc., Chicago, IL, USA) and *p* value <0.05 was considered as statistically significant in all results.

## Results

### Clinicopathologic characteristics of all patients

A total of 233 females were included in this study and their mean age was 50.2 ± 11.0 years. Median follow-up duration was 59.0 ± 25.1 months. Among the 233 patients, 14 patients (6.0 %) were younger than 35 years old. 106 patients (45.5 %) received breast conserving surgery (BCS) and 127 patients (54.5 %) underwent mastectomy.

In terms of histologic types, there were 227 cases (97.4 %) of invasive ductal carcinoma and 6 cases (2.6 %) of invasive lobular carcinoma (ILC). Table 1 shows the clinicopathologic characteristics of all patients.

**Table 1:** Clinicopathologic characteristics in this study

Patient characteristics	Number of patients (%)
Age	
<35	14 (6.0 %)
≥35	219 (94.0 %)
Operation	
BCS	106 (45.5 %)
MRM	127 (54.5 %)
Histology	
IDC	227 (94.4 %)
ILC	6 (5.6 %)
ER	
Negative	73 (31.3 %)
Positive	160 (68.7 %)
PR	
Negative	85 (36.5 %)
Positive	148 (63.5 %)
c-erbB2	
Negative	156 (67.0 %)
Positive	77 (33.3 %)
Histologic grade	
1	72 (30.9 %)
2	101 (43.3 %)
3	55 (23.6 %)
Unknown	5 (2.2 %)
Nuclear grade	
1	39 (16.7 %)
2	130 (55.8 %)
3	59 (25.3 %)
Unknown	5 (2.2 %)
Ki67	
<20	136 (58.4 %)
≥20	97 (41.6 %)
*T* stage	
1	116 (49.8 %)
2	103 (44.2 %)
3	13 (5.6 %)
4	1 (0.4 %)
*N* stage	
0	145 (62.8 %)
1	55 (23.8 %)
2	18 (7.8 %)
3	13(5.6 %)

*BCS* breast conserving surgery, *MRM* modified radical mastectomy, *IDC* invasive ductal carcinoma, *ILC* invasive lobular carcinoma, *ER* estrogen receptor, *PR* progesterone receptor

### Expression of YB-1 in breast cancer cell and relationship between expression of cytoplasmic YB-1 and prognostic factors

Cytoplasmic YB-1 expression in cancer cell was evaluated using IRS. High expressions of YB-1 (score 5–12) were observed in 112 (48.1 %) patients and low expressions (score 0–4) were observed in 121 patients (Fig. 1).

Table 2 presents the relationship between expression of cytoplasmic YB-1 and prognostic factors. High expression of cytoplasmic YB-1 was more frequently observed in ER, PR negative tumor than in positive tumor (*p* < 0.001). Regarding to molecular subtypes, there were more HER2 type tumor and TNBC in the high expression group (*p* < 0.001). High expression of cytoplasmic YB-1 was related with histologic and nuclear grade III tumors (*p* < 0.001). Tumors with high ki-67 were more frequently observed in high expression group (*p* < 0.001). Tumor stage was also significantly related with high cytoplasmic YB-1 expression (*p* = 0.017).

**Table 2:** Comparisons of clinicopathologic results according to expression of YB-1

Patient characteristics	Number of patients (%)		*p* value
	Low (*n* = 121)	High (*n* = 112)	
Age			0.210
<35	5 (4.1 %)	9 (8.0 %)	
≥35	116 (95.9 %)	103 (92.0 %)	
Operation			0.117
BCS	61 (50.4 %)	45 (40.2 %)	
MRM	60 (49.6 %)	67 (59.8 %)	
Histology			0.119
IDC	116 (95.9 %)	111 (99.1 %)	
ILC	5 (4.1 %)	1 (0.9 %)	
ER			<0.001
Negative	18 (14.9 %	55 (49.1 %)	
Positive	103 (85.1 %)	57 (50.9 %)	
PR			<0.001
Negative	28 (23.1 %)	57 (50.9 %)	
Positive	93 (76.9 %)	55 (49.1 %)	
c-erbB2			0.051
Negative	88 (72.7 %)	68 (60.7 %)	
Positive	33 (27.3 %)	44 (39.3 %)	
Molecular subtype			<0.001
Luminal A	64 (52.9 %)	30 (26.8 %)	
Luminal B	41 (33.9 %)	32 (28.6 %)	
HER2 enrich	9 (7.4 %)	25 (22.3 %)	
TNBC	7 (5.8 %)	25 (22.3 %)	
Histologic grade			<0.001
1	52 (44.4 %)	20 (18.0 %)	
2	52 (44.4 %)	49 (44.1 %)	
3	13 (11.1 %)	42 (37.8 %)	
Unknown	4	1	
Nuclear grade			<0.001
1	30 (25.6 %)	9 (8.1 %)	
2	72 (61.5 %)	58 (52.3 %)	
3	15 (12.8 %)	44 (39.6 %)	
Unknown	4	1	
Ki67			<0.001
<20	86 (71.1 %)	50 (44.6 %)	
≥20	35 (28.9 %)	62 (55.4 %)	
*T* stage			0.017
1	70 (57.9 %)	46 (41.1 %)	
2	47 (38.8 %)	56 (50.0 %)	
3	3 (2.5 %)	10 (8.9 %)	
4	1 (0.8 %)	0	
*N* stage			0.070
0	78 (64.5 %)	68 (60.7 %)	
1	33 (27.3 %)	22 (19.6 %)	
2	6 (5.0 %)	13 (11.6 %)	
3	4 (3.3 %)	9 (8.0 %)	

*BCS* breast conserving surgery, *MRM* modified radical mastectomy, *IDC* invasive ductal carcinoma, *ILC* invasive lobular carcinoma, *ER* estrogen receptor, *PR* progesterone receptor, *TNBC* triple negative breast cancer

### Prognosis according to cytoplasmic YB-1 expression

**Fig. 2:**
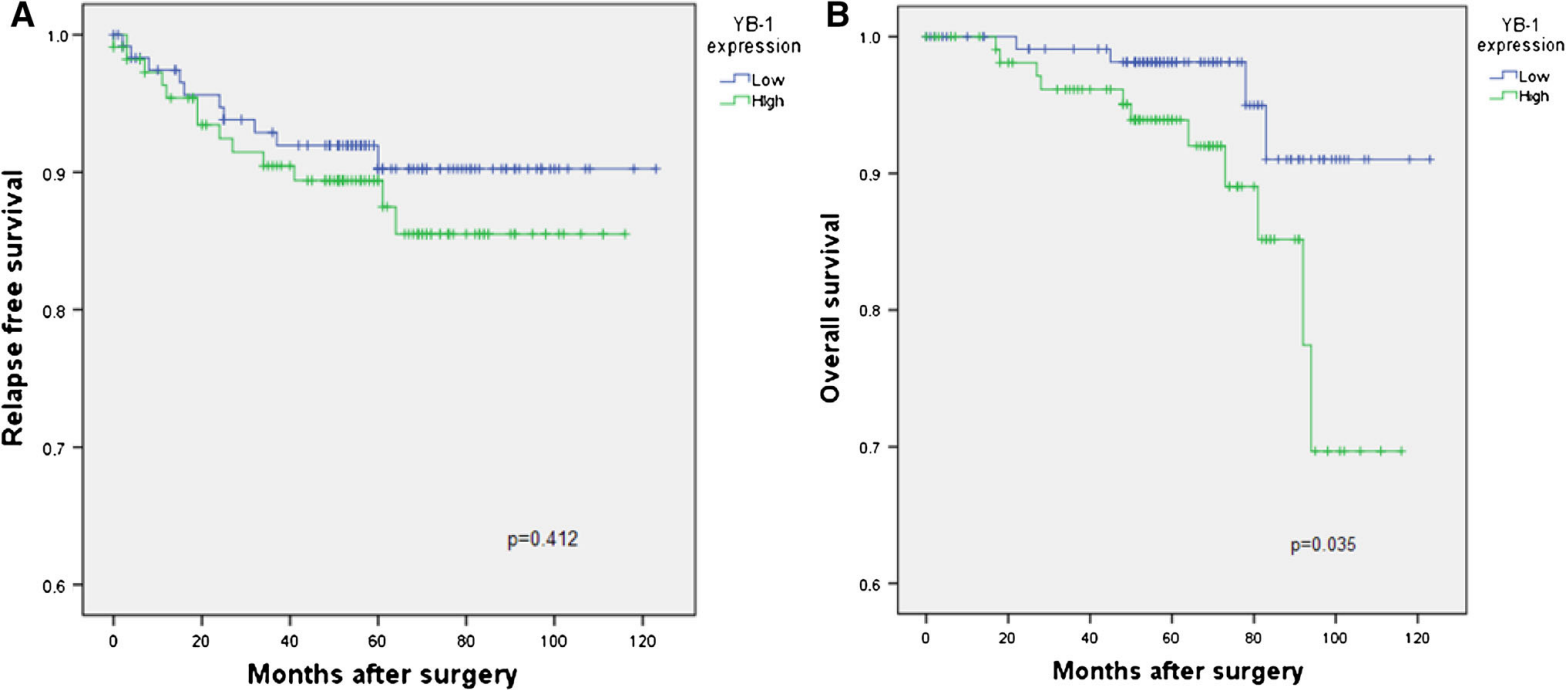
Relapse-free survival and overall survival analysis according to YB-1 expression. **a** Relapse-free survival according to YB-1 expression, **b** overall survival analysis according to YB-1 expression

Kaplan–Meier analysis was conducted to evaluate RFS and OS according to expression of cytoplasmic YB-1. In RFS, there was no significant difference between the two groups (*p* = 0.412) (Fig. 2a). In terms of OS, 5-year survival rates of the high and low group were 92 and 98 %, respectively, showing a significant difference between the two groups (*p* = 0.035) (Fig. 2b). Multivariate analysis was performed using Cox proportionate hazard regression model and high expression of cytoplasmic YB-1 was confirmed as an independent prognostic factor for OS (*p* = 0.043) (Table 3).

**Table 3:** Multivariate analysis for overall survival

	Wald statistic	Degree of freedom	*p* value	Hazard ratio	CI (95 %)
Cytoplasmic YB-1 expression	4.079	1	0.043	3.259	1.035–10.255
Nodal stage	5.411	1	0.020	3.583	1.223–10.501

Including covariates are as follows: age (<35, ≥35), hormonal receptor status (ER/PR), c-erbB2 status, Tumor stage (*T*1 + *T*2/*T*3 + *T*4), nodal stage (*N*0/*N*1 + *N*2 + *N*3), Ki-67 (<20, ≥20)

## Discussion

YB-1 is a DNA- and RNA-binding protein, which is located on chromosome 1p34 and involved in various cellular processes. This protein plays a role in DNA replication, repair, transcription, pre-mRNA splicing, and mRNA translation [3–5]. It was reported that the expression of YB-1 increases in cancer cells and its high expression or expression in nucleus were associated with poor prognosis in many types of cancer [6–9, 13]. Also, this nuclear expression of YB-1 in tumor cell activates gene transcription for protein and relates to multidrug resistance and ionizing radiation [10, 14].

In breast cancer, the oncogenic role of YB-1 has been widely studied. Previous studies showed that the localization of YB-1 in nucleus was associated with poor prognosis as well as with poor prognostic factors [8, 11, 15]. Some studies showed that the nuclear YB-1 expression was positively correlated with the HER2 expression and suggested the inhibition of YB-1 could be a good therapeutic target for HER2 type tumor [15–17]. Ito et al. [18] found that the alteration of YB-1 after neoadjuvant chemotherapy might modify the crosstalk between ER and HER2 pathways, leading to endocrine therapy resistance. According to the study of Li et al. [12], high expression of YB-1 in cytoplasm of cancer cell was significantly associated with poor prognosis in squamous esophageal cancer. However, their study did not show a significant relationship between YB-1 expression and prognostic factors.

In this study, we could observe high cytoplasmic expressions of YB-1 in 112 (48.1 %) patients and YB-1 was also expressed in cancer cell nucleus. Nuclear expression of YB-1 was also significantly related with poor prognostic factors (ER and PR negativity, high histologic and nuclear grade, high Ki-67 expression) and poor OS (data were not shown). However, such an expression was observed in 26 out of 233 cases (11.2 %) and this was a relatively small proportion compared to previous studies [8, 11, 15]. Some previous studies reported that nuclear localization of YB-1 was induced by growth factors and cytokines and related with tumor progression [19–21]. This means that more advanced and aggressive cancer expresses more amounts of nuclear YB-1. In our study, tumors were relatively in their early stages compared to those of previous studies. We consider this finding may have contributed to the lower nuclear expression of YB-1. Actually, among the 26 patients with nuclear expression of YB-1, both nucleus and cytoplasm of YB-1 were observed in 24 patients (99.1 %) and these tumors showed relatively high *T* stage, high histologic and nuclear grade, more aggressive biologic features compared to the tumors expressed YB-1 only in cytoplasm.

Our study showed that high IRS group was significantly related with ER/PR positive, high Ki-67, high histologic grade, high nuclear grade and high *T* stage (*p* < 0.05). Although, c-erbB2 was not significantly related with the high expression of YB-1, there was a tendency that tumor with positive c-erbB2 expressed high YB-1 (*p* = 0.051). The High group was also associated with poor OS (*p* = 0.035), but not with RFS (*p* = 0.412). Subsequently, recurrence patterns according to YB-1 expression were analyzed based on a total of 23 tumor recurrences that were observed. Among them, 13 recurrence cases were observed in the high group and the other 10 recurrence cases in the low group. There were a total of 17 systemic recurrences where 12 (70.6 %) were observed in the high group and 5 (29.4 %) in the low group (*p* = 0.022). These recurrence patterns might be contributing towards the significant difference in OS between the two groups (Table 4). According to previous studies, elevated expression of YB-1 in cancer cell promotes epithelial-mesenchymal transition in various cancers including breast cancer, and this is also related with angiogenesis in tumor microenvironment [22–24]. We think that this mechanism might affect more frequent systemic recurrence and poor OS in the high group. Additionally, during the molecular subgroup analysis for recurrence cases in the high group, three (23.1 %) cases were TNBC, two (15.4 %) were HER2+ type tumors and the rest were luminal type (A or B) tumors. In the low group, there was no TNBC while there was one (10.0 %) and nine cases (90.0 %) of HER2+ type and luminal (A or B) types, respectively. Yi et al. [25] reported that TNBC patients had shorter OS than non-TNBC patients despite favorable response to palliative doxorubicin based chemotherapy. They assumed that this might be due to frequent resistance to chemotherapeutic agent in TNBC. Our prognostic results might be also affected by this mechanism.

**Table 4:** Recurrence patterns according to YB-1

Recurrence pattern	Low group	High group	*p* value
Regional and local	5 (83.3 %)	1 (16.7 %)	0.022
Systemic	5 (29.4 %)	12 (70.6 %)	

There were some limitations of this study. Because our cases were composed of relatively early stage tumors compared to previous studies, there may be fewer cancer related deaths and recurrences compared to other studies [8, 11, 15]. Therefore, this study could not show the independent role of established prognostic factors except the nodal stage.

## Conclusion

This study showed cytoplasmic expression of YB-1 was associated with more aggressive tumor and poor overall survival in breast cancer. Therefore, high cytoplasmic expression of YB-1 in cancer cell could be used as an independent prognostic marker for predicting OS in breast cancer. Furthermore, developing a new targeting agent for YB-1 in cancer cell could be a novel progress in breast cancer treatment.
